# Editorial: Microbial regulation of plant immunity: mechanisms and applications

**DOI:** 10.3389/fmicb.2025.1721914

**Published:** 2025-11-12

**Authors:** Waqar Ahmed, Qaiser Shakeel, Guanghai Ji

**Affiliations:** 1 School of Breeding and Multiplication (Sanya Institute of Breeding and Multiplication), School of Tropical Agriculture and Forestry, Hainan University, Sanya, China; 2 College of Plant Protection, South China Agricultural University, Guangzhou, China; 3 Cholistan Institute of Desert Studies, The Islamia University of Bahawalpur, Bahawalpur, Pakistan; 4 State Key Laboratory for Conservation and Utilization of Bio-Resources in Yunnan, Yunnan Agricultural University, Kunming, China

**Keywords:** plant immunity, microbial regulation, microbiome, disease resistance, sustainable agriculture, biocontrol, omics approaches

In recent years, the role of microbes in regulating plant immunity has emerged as a crucial area of research with far-reaching implications for agriculture, ecology, and biotechnology (Ahmed et al., 2022). The intricate relationship between plants and their associated microorganisms is a cornerstone of plant health and ecosystem stability. The complex relationships between plants and microbes, ranging from beneficial symbiosis to pathogenic interactions, offer unique insights into how plants defend themselves against an array of biotic stressors. This Research Topic explores the diverse and sophisticated mechanisms by which microbes influence plant immune responses, highlighting their potential applications in sustainable agriculture and disease management. For example, the study by Singh et al. on tar spot in corn demonstrates that resistant and susceptible inbred lines harbor distinct phyllosphere microbiomes, with resistant lines associating with beneficial genera like *Cladosporium* and *Quadrisphaera*. This suggests that plant genotype influences microbiome assembly, which in turn, contributes to disease resistance. Similarly, Liu et al. reveal how the antibiotic tetramycin can reshape the soil microbiome, shifting community assembly processes and enriching for beneficial taxa like *Pseudomonas aeruginosa* and *Variovorax paradoxa* to alleviate root rot in *Panax notoginseng*. These studies highlight that manipulating the microbiome, either through host genetics or agrochemicals, is a viable path to enhancing plant health.

Several studies provide deep insights into the molecular mechanisms through which microbes prime plant immunity. Zhang et al. showed that the protein elicitor PeVn1 from *Verticillium nonalfalfae* activates MAPK signaling, calcium ion flux, and the ethylene pathway in strawberry, leading to resistance against *Botrytis cinerea*. At the genetic level, Ishfaqe et al. identified and characterized the *NPR1* gene family in chili pepper, linking its expression to resistance against begomoviruses. Furthermore, Luo et al. comprehensively review the role of microRNAs (miRNAs) as master regulators of defense-related genes, even highlighting their potential for cross-kingdom RNA silencing to inhibit pathogens. These findings underscore the complexity of the immune signaling network and the multiple entry points through which microbes can modulate it. Similarly, it is reported that plant disease management can also be achieved by the application of specific microbes as biocontrol agents (Ayaz et al., 2023). For example, Fu et al. and Yeo et al. demonstrates the potent antifungal activity of *Bacillus velezensis* against *Colletotrichum fioriniae* and *Fusarium* head blight, respectively, with the latter also inducing systemic resistance in rice. Yang et al. identified oxalic acid from *Aspergillus tubingensis* as a novel nematicidal compound that also strengthens root lignification. The fight against nematodes is further advanced by Ayaz et al. which reviews the promise of multi-omics approaches for discovering new BCAs and nematicides. The successful biocontrol of diseases in high-value crops like *Morchella esculenta* Chen, Zhang et al. and chili pepper Iqbal et al. reinforces the practical viability of these microbial solutions.

A complete picture of plant immunity requires understanding the offensive strategies of pathogens. Chen, Li et al. provides a comprehensive overview of how plant-parasitic nematodes suppress host defenses using effector proteins, while Aslam et al. explore the emerging role of epigenetic modifications in the pathogenicity of *Magnaporthe oryzae*. These studies confirmed that the battle between plant and pathogen is a co-evolutionary arms race, and durable resistance strategies must account for pathogen adaptability. Qian et al. employed the integrated transcriptomics and metabolomics technique. They pinpointed key flavonoids such as naringenin, kaempferol, and quercetin involved in the defense of *Morus notabilis* against ring rot. Similarly, Habib et al. employ genome-wide association studies (GWAS) in quinoa to link panicle architecture with yield, a trait inherently linked to plant health. These omics-driven approaches are indispensable for unraveling complex traits and identifying critical genes and metabolites for crop improvement. Overall, this Research Topic unequivocally establishes that microbial regulation is a central pillar of plant immunity. From the leaf surface to the rhizosphere, beneficial microbes act as key allies, modulating host physiology and suppressing pathogens through a multitude of mechanisms. Thus, we conclude that by continuing to decode the complex language of plant-microbe interactions, we can harness this knowledge to develop robust, sustainable, and productive agricultural systems for the future.
